# Adenosine kinase as a therapeutic target in traumatic optic neuropathy

**DOI:** 10.1186/1471-2164-15-S2-P58

**Published:** 2014-04-02

**Authors:** Saif Ahmad, Nehal El-Sherbiny, Ahmed El-Sherbini, Sadan Fulzele, Gregory I Liou

**Affiliations:** 1 Department of Biological Sciences, Rabegh College of Science and Arts, King Abdulaziz University, Rabegh Campus, Saudi Arabia; 2 Department of Ophthalmology, Georgia Regents University, Augusta, GA, USA; 3 Faculty of Pharmacy, Mansoura University, Mansoura, Egypt; 4 Department of Orthopedics, Georgia Regents University, Augusta, GA, USA

## Background

The purpose of this study is to understand the mechanism of traumatic optic neuropathy (TON) in order to prevent vision loss. Following traumatic insults to the optic nerve, retinal microglia cells are activated through MAP Kinase pathways and increased cytotoxic activity that causes retinal ganglion cell death [1]. Under stress condition, extracellular concentration of adenosine is likely to increase and activates an anti-inflammatory pathway through A2A adenosine receptor. But in TON, the accumulated extracellular adenosine is then transported intracellular through equilibrative nucleoside transporters (ENTs) which further gets converted into AMP by Adenosine kinase (AK), which results in low extracellular adenosine concentration. We have demonstrated that microglia activation and TNF-α release was inhibited by AK inhibitor. Based on these findings, we hypothesize that an imbalance in adenosine formation and metabolism in the retinal microglia participated by AK may contribute significantly to retinal complications in the setting of TON.

## Materials and methods

Mice were anesthetized according to standard protocol and bilateral limbal conjunctival peritomy was performed posteriorly to the optic nerve in each mouse. Mice were treated with or without an AK inhibitor (AKI), ABT702 (25μg/kg; i. p.), every other day for 7 days. All retinas or eye ball were then harvested for protein, histology and RNA expression.

## Results

**Figure 1:**
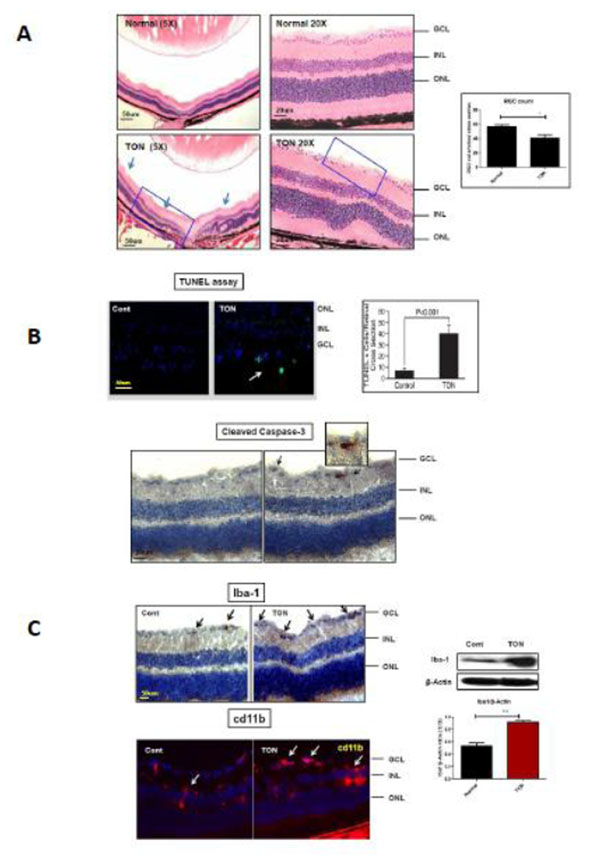
**A)** H & E staining of TON eye section for RGC counting. **B)** Quantitative analysis of TUNEL-positive cells in the retinal cross section of TON and control. Immunohistochemical analysis of activated Caspase-3 in retinal section of TON vs. control. **C)** Immunohistochemical and Immunoblotting of microglial activation marker Iba-1 and CD11b expression in TON vs. control.

We found that in TON, RGC number significantly decreased, Cleaved Caspase-3, Iba1, cd11b expression and TUNEL positive cells increased as compared with control eye (Fig 1 A-C). In a series of experiments with Western Blot, Immunohistology and Real-Time PCR, increased expression of TNF-α, Iba1, iNOS and IL-6 were shown in nerve crush model as compared with AKI treatment. It was also noted that administration of AKI showed no effect in the control.

## Conclusions

Based on our findings, we conclude that Adenosine Kinase inhibition may have potential role in regulating inflammatory mechanism in TON mouse model.
